# Long-term adaptive laboratory evolution of *Saccharomyces cerevisiae* for high-titer lactic acid production at low pH

**DOI:** 10.1186/s12934-025-02867-x

**Published:** 2025-11-26

**Authors:** Martin Altvater, Irene Tomico-Cuenca, Diethard Mattanovich, Michael Sauer

**Affiliations:** 1 Institute of Microbiology and Microbial Biotechnology, Department of Biotechnology and Food Science, BOKU University, Muthgasse 18, Vienna, 1190 Austria; 2 Austrian Centre of Industrial Biotechnology (ACIB) GmbH, Vienna, Austria; 3 Present Address: OMV AG, Trabrennstraße 6-8, Vienna, 1020 Austria

**Keywords:** Lactic acid production, Adaptive laboratory evolution, Tolerance to low pH, Industrial biotechnology, Saccharomyces cerevisiae

## Abstract

**Background:**

Lactic acid is a highly versatile molecule whose increasing demand across the polymer, food, pharmaceutical, chemical, and cosmetics industries underscores its industrial and economic significance. Currently, lactic acid is predominantly produced via microbial fermentation using lactic acid bacteria facing limitations such as sensitivity to low pH, complex nutritional requirement and waste product generation during downstream processing.

**Results:**

To address these challenges, we employed a genetically modified *Saccharomyces cerevisiae* strain capable of producing lactic acid and subjected it to long-term adaptive laboratory evolution. The strain was cultured in serial shake flask cultivations over a period of 35 months under elevating lactic acid concentrations and increasing stress to low pH. The evolved populations showed improved production of up to 250% in final lactic acid titers compared to the parental strain. The best-performing strains reached 67 g L⁻¹ at a final pH of 2.4 without pH control or 165 g L⁻¹ lactic acid at pH 3.0 with the addition of pH neutralizers, representing - to our knowledge - the highest LA titer reported in shake flask cultivations for *S. cerevisiae.*

**Conclusion:**

Overall, our results prove the great potential of long-term adaptive laboratory evolution in developing robust yeast cell factories for industrial organic acid production.

## Background

Lactic acid (LA) is a very versatile molecule and finds application in the polymer, food, chemical, pharmaceutical, and cosmetics industry [1]. The global demand for LA production is constantly rising, primarily driven by the growing need as building block for the biodegradable polymer polylactic acid (PLA) and thus makes LA an important molecule for industrial production [2–5]. Although precise industrial figures are difficult to obtain, estimations within the field suggest that LA is currently mainly produced by microbial fermentation, with bacteria (e.g. lactic acid bacteria (LAB)) representing the prevalent production hosts. Due to high yield, productivity, and acid tolerance resulting in >200 g/L lactic acid titers in production processes, LAB play an important role in industrial biotechnology [5–8].

However, there are several disadvantages of using LAB for industrial LA production and purification. Most LAB require organic nitrogen sources necessitating the addition of complex supplements in the fermentation medium. These additives not only raise production expenses but also complicate the downstream purification of LA [9]. Moreover, the majority of LAB generate both isomers, D- and L-LA making it more challenging and expensive to isolate a pure enantiomer [10]. Furthermore, LAB are sensitive to acidic environments, requiring pH control with neutralizing agents such as calcium carbonate. During production, this results in the formation of calcium lactate, from which LA must later be recovered through acidification using sulfuric acid, leading to equimolar formation of calcium sulfate (gypsum) - a problematic byproduct both economically and environmentally. For every ton of LA produced, roughly one ton of gypsum is generated, complicating downstream processing and increasing waste disposal costs [1, 4].

To overcome these limitations, a key strategy of researchers in recent years is to produce LA at low pH, where the acid remains in its undissociated form. Given that the pKₐ of LA is 3.86, lowering the pH significantly shifts the dissociation equilibrium. For example, at pH 4.5 only 19% of LA is protonated compared to 88% at pH 3.0. This shift reduces the requirement for neutralizing agents and consequently the formation of gypsum, facilitating purification and reducing costs.

In recent years, yeasts have gained attention as alternative hosts for LA production. Yeast species such as the industrially well-established *Saccharomyces cerevisiae*, offer advantages over LAB such as inherent tolerance to low pH conditions and minimal nutritional requirements leading to easier downstream processing of the products [1, 11–13]. Various yeast species have been genetically engineered for rerouting their native fermentation pathways towards an enantiomerically pure D- or L-LA and thus form a very promising group of organisms for future industrial LA production (summarized in [5]) and first companies are developing and employing yeasts as production hosts, proving the potential of yeasts as alternative cell factories for industrial LA production [14]. Although yeasts are generally more tolerant to acidic environments than LAB, LA still imposes substantial stress on the cells [12, 15]. For that reason, adaptive laboratory evolution (ALE) has emerged as a powerful complementary approach, to rational genetic engineering, to improve tolerance to LA and further enhance LA production at low pH [16, 17].

Previous ALE studies with yeast strains have yielded significant but still mild improvements in acid tolerance and LA production, these were achieved after a relatively short evolution time [18–20]. In contrast, we hypothesized that long-term ALE over years offers the potential for more substantial physiological adaptations through gradual accumulation of beneficial mutations under sustained selective pressure. In our study, we subjected a genetically modified *S. cerevisiae* [21] to a long-term ALE over 35 months under increasing LA and low pH stress with the goal of developing a robust and high-performing LA production yeast cell factory for an efficient, low pH, and neutralizer-low/free L-LA production process.

## Materials and methods

### Strains

The engineered non-evolved parental *S. cerevisiae* strain LACp described in Totaro et al. 2020 [21] (strain background: diploid CBS 7962) was used as strain subjected to ALE. The strain contains an episomal 2µ-high-copy plasmid expressing the *URA3* gene as auxotrophic marker, an hph (hygromycin B phosphotransferase) resistance cassette enabling antibiotic selection with 300 µg/mL hygromycin B added every 2–3 weeks to the evolution cultures to maintain plasmid stability, and the lactate dehydrogenase gene (LDH) of *Lactiplantibacillus plantarum* under the control of the yeast *TPI1*-promoter. To avoid carbon flux from the substrate glucose to any other metabolites than towards LA, the three major isoforms of the pyruvate decarboxylase genes (*PDC1*,* PDC5*,* PDC6*) were deleted. The resulting strain is unable to grow on glucose as sole carbon source due to the deletions of the pyruvate decarboxylase genes [22]. A C_2_-source, in this work ethanol, was added as an additional carbon source to the media allowing the strain to generate the essential cytosolic acetyl-CoA and biomass.

### Media

**Fig. 1:**
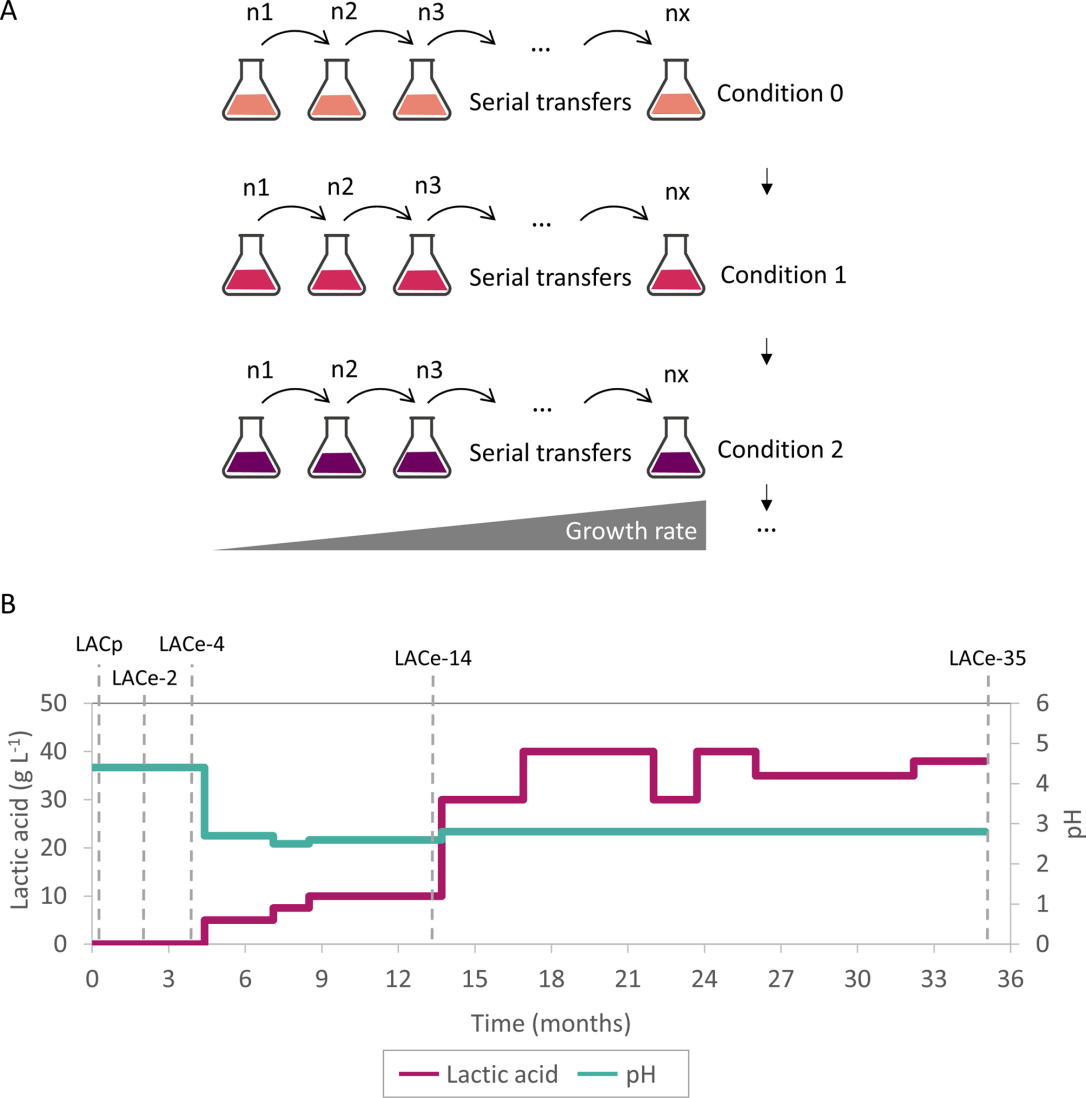
Long-term adaptive laboratory evolution of *S. cerevisiae* for enhanced tolerance to low pH and high lactic acid concentrations. (A) Overview of ALE strategy; (B) LA concentration and initial pH level of evolution medium composition over 35 months of ALE

The medium for ALE consisted of 100 g L^− 1^ (0–4 months of ALE) and 150 g L^− 1^ glucose (month 5–35), 5 g L^− 1^ ethanol, 4.54 g L^− 1^ urea, and 3.4 g L^− 1^ yeast nitrogen base without amino acids and ammonium sulfate (YNB), adjusted to the described pH with HCl or NaOH and increasing LA concentrations over 35 months (0–38 g L^− 1^ LA, Fig. 1B). Seed culture medium consisted of 10 g L^− 1^ ethanol, 4.54 g L^− 1^ urea, and 3.4 g L^− 1^ YNB, initial pH 4.5. Production medium consisted of 150 (for production experiments with start OD_600_ = 2) or 200 g L^− 1^ glucose (for production experiments with start OD_600_ = 50, for potential LA titers > 150 g L^− 1^), 5 g L^− 1^ ethanol, 4.54 g L^− 1^ urea, 3.4 g L^− 1^ YNB, initial pH 4.5, and 0–35 g L^− 1^ CaCO_3_. CaCO_3_ was heat sterilized in shake flasks previous to the addition of the production medium.

### Adaptive laboratory evolution

The production strain was cultured and diluted in 10 mL ALE medium in 100 mL shake flasks every 24 h to an initial OD_600_ of 0.5 (spectrophotometer: WPA CO 8000 Biowave Cell Density Meter), and cultured at 30 °C and 180 rpm creating aerobic conditions. When longer incubations (48–72 h) were necessary, e.g. over weekends, cultures were diluted to an appropriate lower OD_600_ value to avoid cells reaching the stationary phase before the next dilution step. Evolution was started with four (A, B, C, D) parallel populations under the same conditions and tested for their LA production capability at different time points throughout the course of the evolution. Populations not showing improvement in growth rate or LA production were discontinued. Cultures A and B were stopped after two and four months, respectively. Population D was split after two months into two cultures before both were discontinued after four and five months, respectively. Population C showed the best development throughout the whole evolution experiment and was cultured for 35 months, corresponding to 3500–4000 generations (2–5 generations per 24 h).

### Lactic acid production

Strains from ALE at different time points (months of ALE: 0, 2, 4, 14, 35; strains named accordingly: LACp (p: parent), LACe-2 (e-2: evolved for 2 months), LACe-4, LACe-14, LACe-35) were analyzed for their LA producing capability in batch shake flask cultures under the described conditions. Seed cultures were grown in 500 mL in 5 L shake flasks at 30 °C and 180 rpm for 2 days, pelleted (1,500 g, 5 min) and resuspended in 20 ml production medium to the indicated OD_600_ in 100 mL shake flasks (for cultures with start OD_600_ = 2) or 250 mL shake flasks (experiments with start OD_600_ = 50) containing sterilized 0–35 g L^− 1^ CaCO_3_ for pH regulation and incubated at 30 °C and 180 rpm for 2–5 days. Glucose and LA concentrations were analyzed by HPLC analysis by using a method previously developed in our laboratory [23]. To maintain comparability with earlier studies in the research field, yield calculations did not account for evaporation losses in the shake flask cultures.

## Results and discussion

### Adaptive laboratory evolution

The parental starting *S. cerevisiae* strain LACp was engineered by introducing a plasmid harboring a heterologous lactate dehydrogenase from *Lactiplantibacillus plantarum* to convert pyruvate directly to LA, while eliminating the competing ethanol formation pathway through deletion of pyruvate decarboxylases *PDC1*, *PDC5*, and *PDC6* [21]. This genetic modification redirected the carbon flux from glucose toward LA production, however, the elimination of pyruvate decarboxylase activity rendered the yeast dependent on an exogenous C_2_ source for the essential cytosolic acetyl-CoA formation. In this work, ethanol was provided as C_2_ molecule. This strain was subjected to adaptive laboratory evolution in shake flasks by diluting the yeast populations almost daily. The initial selection pressure was adjusted so that cells doubled twice in 24 h under stress (Fig. 1A, condition 0). As evolved populations demonstrated increased growth rates over weeks at the particular condition - achieving four to five doublings within 24 h (µ = 0.12–0.14 h^− 1^) - the medium composition was changed to impose higher selective pressure (Fig. 1A, condition 1) reducing the growth rate of the first populations cultured in the new condition to two doublings in 24 h (µ = 0.06 h^− 1^), improving over time. These iterative cycles were repeated over a total period of 35 months (Fig. 1A, condition 2 etc.).

The specific levels and changes of the medium composition are shown in Fig. 1B: The evolution medium contained the indicated concentrations of glucose (first 100 g L^− 1^, later adjusted to 150 g L^− 1^), ethanol (constant 5 g L^− 1^), and the main stressors LA (0–38 g L^− 1^) and a low pH environment (pH 4.5–2.6). The evolution medium from month 0 to month 4 was adjusted to pH 4.5 and no LA was added (Fig. 1B). During this period, the only LA and acidification stress originated from the produced LA of the evolving yeast population itself, reaching up to 5–8 g L^− 1^ LA in the 24 h cultures and lowering the pH to around 3.0–2.8 (data not shown). After 4 months, the strains gained sufficient robustness to tolerate additional stress. External LA was then gradually added to the medium, while the initial pH was lowered to 2.8 and, due to continued LA production of the cells, further declined to around 2.4 over the course of 24-hour incubations (data not shown). Several shake flask populations were evolved in parallel cultivations and tested and compared throughout the evolution at different time points for their LA production capability at low pH. It is important to note that the progression of evolution was not always linear. Populations not showing significant improvements in growth rate or LA production during the early phase of the evolution (until month 5) - when mutations with large and pleiotropic fitness effects typically occur [24, 25] - were consequently discontinued. This underlines the inherent variability in ALE and the necessity for continuous monitoring and selection during an ALE process.

### Lactic acid production across evolved strains

**Fig. 2:**
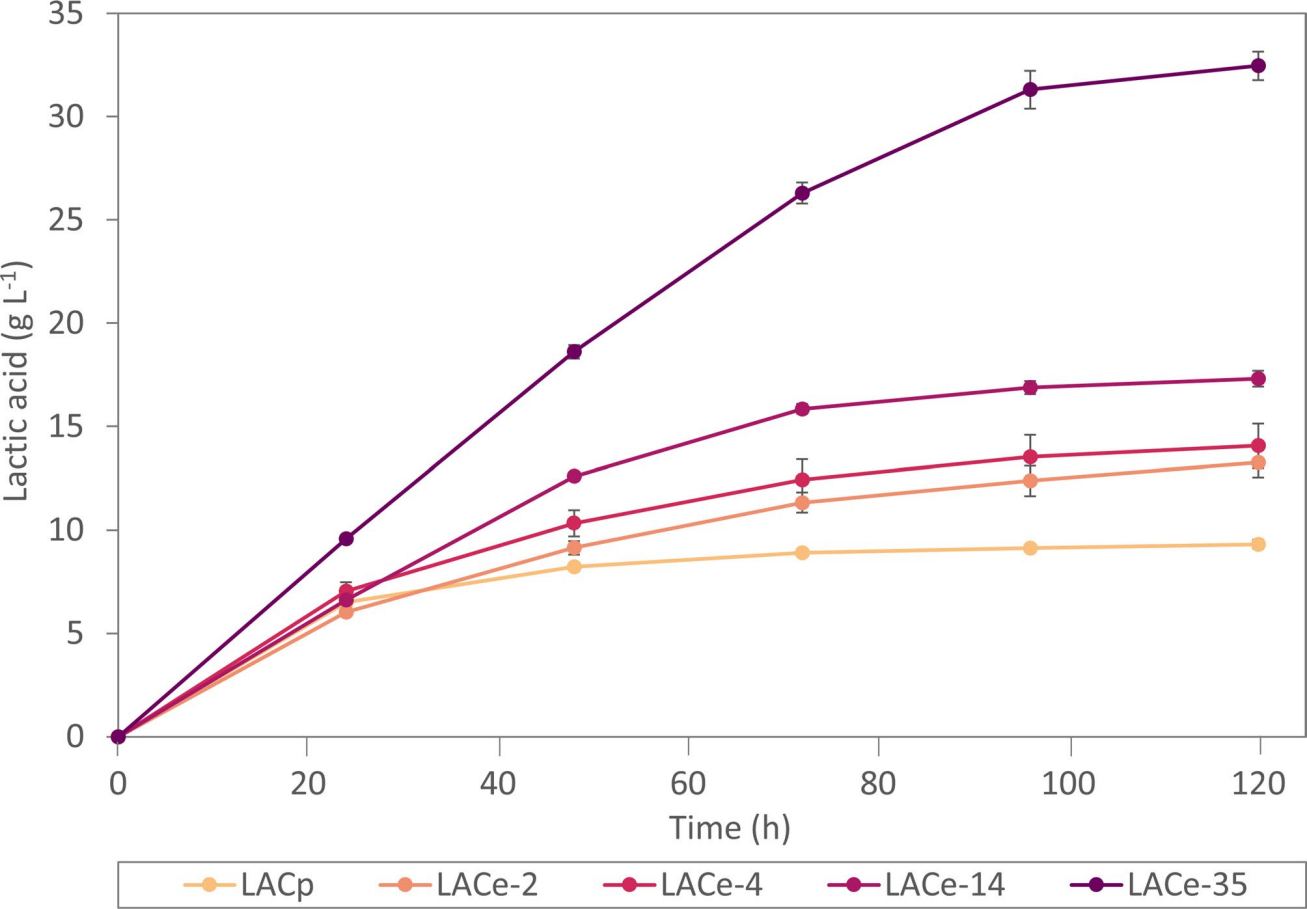
Comparison of LA production of differently long evolved populations. 0 g L^−1^ CaCO_3_; OD_600_ = 2; values represent the mean ± standard error of three biological replicates

Figure 2 shows a comparison of populations of differently evolved cells (0, 2, 4, 14, 36 months, named LACp, LACe-2, LACe-4, LACe-14, LACe-35, see also dashed lines in Fig. 1B) in a LA production experiment without any pH control and with a start OD_600_ of 2, resembling the conditions of the ALE. Comparison of the production reveals that strains with longer exposure to evolution conditions produced higher LA titers, reflecting gradually acquired tolerance to low pH and high LA concentrations over the course of 35 months of evolution. For instance, after 4 months and about 350 generations, production of LA improved by 48% from 9.3 g L^− 1^ (parental strain LACp) to 13.5 g L^− 1^ (LACe-4) during 120 h production time. Strain LACe-35, evolved for 35 months and over 3500 generations, reached 32.5 g L^− 1^ of final LA titer, representing an improvement of 250% in comparison to the non-evolved parental strain LACp. Final pH values for all the cultures were between 2.4 and 2.5 (data not shown).

### Influence of pH on lactic acid production

The production experiments presented in Fig. 2 were conducted without pH neutralizers which would counteract the acidification by the LA formation. However, in industrial setups there are scenarios where controlling the pH by adding chemical compounds such as CaCO_3_ can be favorable. While higher pH levels can create a less toxic environment for the cells leading to improved substrate-to-product conversion and increased LA titers, low pH values are beneficial for a cost-effective downstream processing and reduced by-product formation. Consequently, a potential industrial process starts at higher pH levels for ideal product formation and is operated so that by the end of the production, the medium reaches a pH well below the pK of lactic acid of 3.86 for efficient product recovery of the free acid [26].

**Fig. 3:**
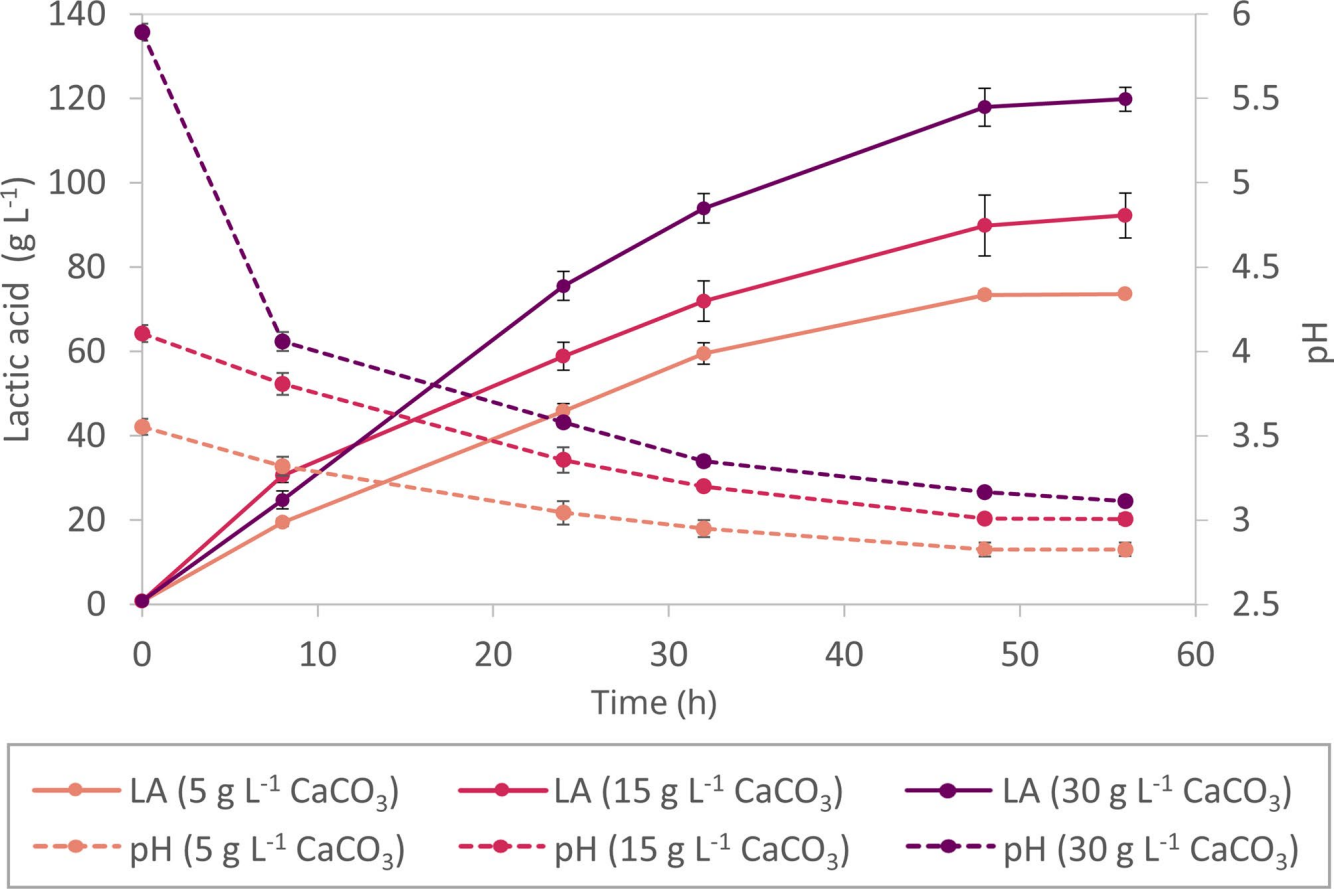
Impact of pH regulation on LA production. 5, 15, or 30 g L^−1^ CaCO_3_; strain LACe-35; OD_600_ = 50; values represent the mean ± standard error of three biological replicates

To investigate the dependency between pH and LA production in our setup, varying concentrations of CaCO_3_ (5, 15, 30 g L^− 1^) were added to production cultures of the most evolved strain, LACe-35. While we focus on developing cells obtaining high final LA titers at low pH with our ALE approach at the biological scale, we address production rates at the bioprocess level with high biomass inocula. The initial high cell density of OD_600_ of 50 further limits oxygen supply to create microaerobic conditions ideal for lactic acid production [27, 28]. The results presented in Fig. 3 show a clear correlation between CaCO₃ concentration (and thus pH) and both the productivity and final LA, aligning with previous studies [20, 29]. Specifically, the addition of 5 g L^− 1^ CaCO_3_ resulted in a final LA titer of 75 g L^− 1^ at pH 2.8; 15 g L^− 1^ CaCO_3_ yielded 92 g L^− 1^ LA at pH 3.0; and 30 g L^− 1^ CaCO_3_ led to 120 g L^− 1^ LA at final pH 3.1. These results highlight the enormous impact of pH regulation on LA production.

### Influence of ALE conditions on LA production

Following the assessment of pH regulation on LA production at defined production conditions, the next step was to investigate how different ALE durations and conditions influence LA production at varying pH environments. Specifically, we compared yeast strain LACe-4 evolved for 4 months at relatively moderate pH levels (pH range 4.5–2.8, month 0–4, Fig. 1B) to the most evolved strain LACe-35 in very low pH conditions (pH 2.8–2.4, month 5–35) to assess how their performance differs depending on the pH environment.

**Fig. 4:**
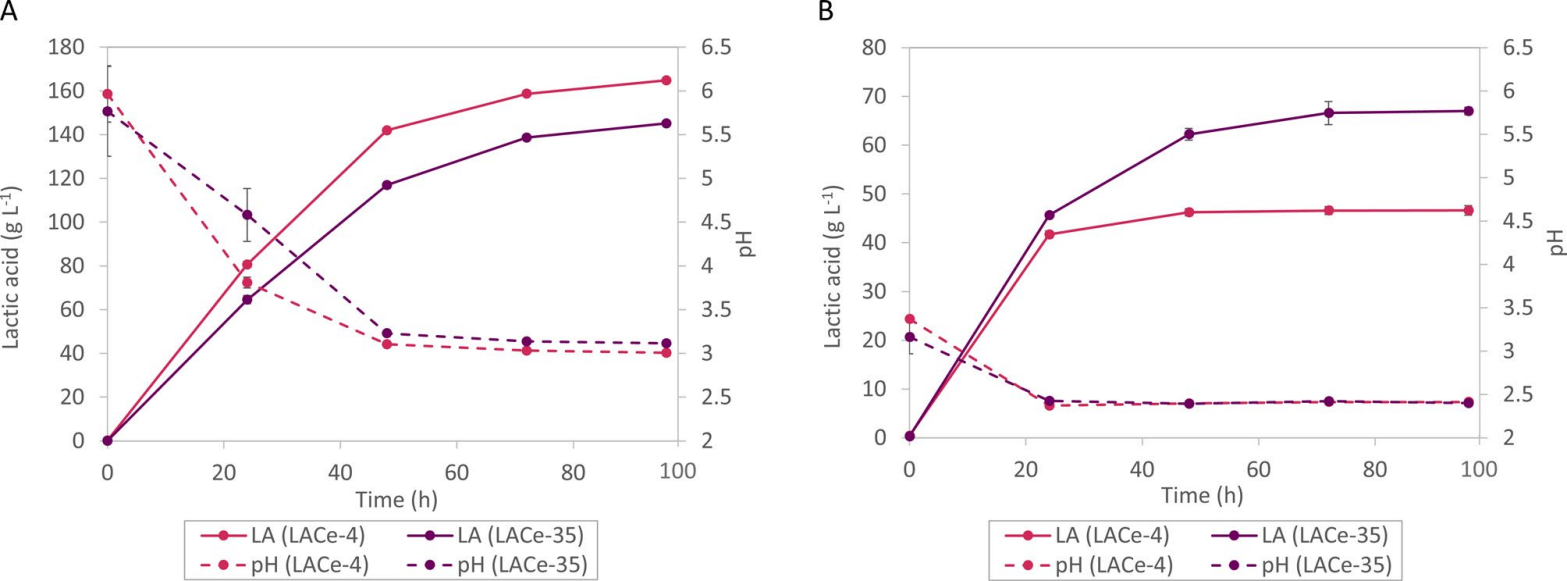
Comparison of LA production of LACe-4 and LACe-35 at different pH conditions. (A) Moderate pH levels: 35 g L⁻¹ CaCO₃; OD_600_ = 50; (B) low pH levels: 0 g L⁻¹ CaCO₃; OD_600_ = 50

To mimic two distinct industrial scenarios, shake flask cultivations were designed under conditions optimized for maximum LA titer or favoring the lowest final pH possible. In the high-titer scenario, 35 g L^− 1^ CaCO₃ - the highest concentration tolerated in our setup before calcium lactate precipitation occurred at later fermentation stages (data not shown) - was added to the cultures (Fig. 4A). At these conditions, the LACe-4 strain outperformed the longer-evolved LACe-35, reaching 165 g L^− 1^ LA at pH 3.0 with a yield of 0.89 g g^− 1^ LA per consumed glucose, compared to 145 g L^− 1^ at pH 3.1 for LACe-35. To our knowledge, this represents the highest LA titer reported for *S. cerevisiae* in shake flask cultivations [5]. In contrast, at conditions without any pH neutralizer (Fig. 4B), the LACe-35 strain showed superior performance, producing 67 g L^− 1^ LA with a final pH of 2.4 and a yield of 0.75 g g^− 1^, compared to 46 g L^− 1^ LA and pH 2.41 of LACe-4 in this low-pH environment. Table 1 summarizes the LA production of the two top-performing strains at the particular pH condition.

Defining universal conditions optimal for industrial production is challenging as they depend on company-specific economic analyses and process requirements, particularly for the purification. Costs are the decisive factor for setting up an industrial production process. The product yield obtained by the strains decides about the substrate costs and should therefore be as high as possible. Our strains obtain 0.89 g LA per g glucose at final pH 3 (Table 1). The second most important cost factor is the purification of the LA - particularly when polymer grade is required. Here, a final compromise has to be found if a somewhat lower titer is acceptable when the final pH is lower, or if by addition of small amounts of neutralizer the pH is kept somewhat higher and therefore the titer is higher. The optimal choice ultimately depends on the setup of the individual purification process. Our strains are optimal to design such a process. The production rate can be influenced by the amount of biomass used - high cell density cultures show high volumetric productivities and the pair titer versus pH can be adapted to fulfill the purification needs.

Taken together, the results indicate that each strain performs optimally under conditions resembling those of its respective evolution history. LACe-4, evolved at moderate pH levels, excels in environments with higher pH (Fig. 4A), while LACe-35 demonstrates superior productivity at highly acidic conditions (Fig. 4B). These findings underscore the importance of aligning evolutionary conditions with targeted process environments to maximize microbial production performance, and highlight the potential of long-term ALE to develop robust yeast cell factories for low-pH industrial production processes.

**Table 1:** Experimental values of cultivations presented in Fig. 4

Strain	LA (g L^− 1^)	Glucose consumed (g L^− 1^)	Yield (g g^− 1^)	Final pH	CaCO_3_ (g L^− 1^)
**LACe-4**	**164.89 ± 1.04**	**185.54 ± 2.09**	**0.89 ± 0.01**	**3.01 ± 0.03**	**35**
LACe-35	145.18 ± 0.95	162.93 ± 1.62	0.89 ± 0.01	3.12 ± 0.01	35
**LACe-35**	**67.00 ± 0.77**	**89.23 ± 2.22**	**0.75 ± 0.02**	**2.40 ± 0.02**	**0**
LACe-4	46.63 ± 0.97	70.74 ± 2.64	0.66 ± 0.03	2.41 ± 0.01	0

Best LA production at individual pH conditions is highlighted in bold; values represent the mean ± standard error of three biological replicates

## Conclusion and outlook

In this work, we successfully applied long-term adaptive laboratory evolution to enhance the LA production capacity of a genetically engineered *S. cerevisiae* strain under low pH conditions. Consistent with previous findings, our results showed that noticeable evolutionary improvements can emerge after just a few hundred generations. However, extended evolution over 35 months and more than 3500 generations resulted in robust yeast cells producing substantially higher LA titers and at very low pH levels. Production conditions with pH values down to 2.4 are very attractive in industrial settings as, at such a low pH, 97% of the LA molecules are present in the undissociated form facilitating downstream purification, avoiding waste product formation, and thus reducing time and costs. These findings validate long-term ALE as powerful tool for developing robust microbial cell factories and highlight the potential of yeast as an alternative production host for industrial organic acid biosynthesis, overcoming key limitations associated with traditional bacterial fermentation systems.

Future work will focus on scaling up fermentation processes to bioreactor systems providing insights into the industrial feasibility of the evolved yeast strains. Genome sequencing and analysis of the mutations acquired during evolution may uncover new targets for further strain improvements by rational metabolic engineering. Ultimately, this research helps pave the way for more efficient and sustainable organic acid production contributing to the development of greener industrial biotechnological processes.

## Data Availability

Not applicable.
